# Estimating Amino Acid Requirements in Real-Time for Precision-Fed Pigs: The Challenge of Variability among Individuals

**DOI:** 10.3390/ani11123354

**Published:** 2021-11-24

**Authors:** Aline Remus, Luciano Hauschild, Marie-Pierre Létourneau-Montminy, Candido Pomar

**Affiliations:** 1 Sherbrooke Research and Development Centre, Agriculture and Agri-Food Canada, Sherbrooke, QC J1M 0C8, Canada; 2 Department of Animal Science, School of Agricultural and Veterinarian Sciences (FCAV), University of São Paulo State (UNESP), Jaboticabal 14884-900, São Paulo, Brazil; luciano.hauschild@unesp.br; 3 Department of Animal Science, Faculty of Agriculture and Food Sciences, Laval University, Quebec City, QC G1V 0A6, Canada; marie-pierre.letourneau-montminy.1@ulaval.ca

**Keywords:** precision feeding, precision nutrition, precision livestock farming

## Abstract

**Simple Summary:**

Precision feeding considers the difference in amino acid requirements among pigs and over time by providing daily tailored diets. This practice allows improving environmental and economic performances. Future systems should focus on maximizing nutrient use efficiency to move towards “green” pig production. This study explored a new method of providing amino acids to maximize their use, mainly focusing on understanding variations in the protein metabolism response among individuals to minimize variation in growth response. This study showed that even pigs fed the same amino acid level might use these nutrients differently, especially in protein deposition. Logically, pigs with the greatest protein deposition are the ones that use amino acids the most efficiently, thus exhibiting the lowest nitrogen excretion. This study helped identify some of the factors affecting the efficiency of nitrogen use in pigs. By improving the understanding of pigs’ nutrient response, pig production can become more resource-efficient.

**Abstract:**

This study aimed to measure protein deposition (PD) in pigs fed with daily tailored diets where either dietary lysine (Lys) or threonine (Thr) were provided at independent levels (ignoring an ideal ratio). A total of 95 growing pigs (35 kg body weight (BW)) with electronic ear tags granting them access to automatic feeders were randomly assigned to treatments. The setup was an unbalanced 2 × 5 factorial arrangement with Lys and Thr provided at five levels (i.e., 60%, 80%, 100%, 120%, and 140% of the estimated individual requirements of Lys and Thr), resulting in 25 treatments for 21 days. The observed PD variation to Lys and Thr provisions was large, with Lys and Thr intake explaining only 11% of the variation. Cluster analysis discriminated pigs with low (167 g/d, *n* = 16), medium (191 g/d, *n* = 38), and high (213 g/d, *n* = 37) PD, but with a similar amino acid intake. Differences in PD were associated with differences in nutrient efficiency of utilization. Providing Lys and Thr in a factorial mode, ignoring an ideal ratio, did not decrease the variability in PD. Future research efforts should focus on identifying and investigating the sources of interindividual variability—a necessary step before final recommendations can be made for AA in precision-fed pigs.

## 1. Introduction

The nutrient requirements of a pig population can be defined as the amount of nutrients needed to achieve specific production objectives such as maximizing weight gain and lean tissue gain and improving feed conversion. Nutritional requirements might vary according to body weight, health status, genetics, and sex, among other less known factors. Most commonly, lysine (Lys) requirements are estimated as a function of the average daily feed intake (ADFI), average daily gain (ADG), body weight (BW), and the potential for protein deposition (PD). Meanwhile, all other amino acids (AA) requirements are estimated using a fixed AA to Lys ratio [1,2,3] identified as the ideal protein profile. In almost all methods used to estimate AA requirements, it is assumed that for a pig in good health with known BW and protein deposition potential, PD is determined by the amount of nutrients consumed, without any variation among pigs. Traditional mathematical models [1,2] use the average pig to estimate the AA requirements of pigs raised in large groups and fed the same feed for extended periods (i.e., phase-feeding). Therefore, the variance among individuals and over time is ignored [4]. However, within a population, pigs with similar nutrient intake vary greatly in growth response [5,6].

Individual pigs have specific nutrient requirements that vary over time [7]. Establishing AA requirements can be hampered by several factors that contribute to increased variability in the response of individual animals. It was previously found that daily requirements for Lys vary among individual pigs [8,9,10]. Estimating real-time individual AA requirements is challenging given that pigs with similar AA provisions [11] or with similar BW [12] showed large variation in PD. Similar PD variation has been found within treatments in many dose–response studies [6,13,14]. In these studies, the test AA is provided at different levels, ranging from excess to deficiency, and Lys is the reference AA and is provided at a low limiting level (e.g., 90% of the estimated requirements), whereas all the other AA are provided in excess (e.g., >110% of the known requirements) [15]. However, the individual [16] and group [17] pigs’ growth response to AA intake varies significantly in these studies, and it can be hypothesized that the dose–response methodology increases growth response variance because some pigs receive limiting amounts of AA. Therefore, a factorial approach was proposed to independently estimate real-time Lys and Thr requirements of pigs fed daily with daily tailored diets. In contrast to the traditional approach used in dose–response studies, which assumes that AA is required in a fixed ratio to Lys, this novel approach allows estimating two AA at a time without limiting any other AA in the diet other than the test AA. This novel approach also allows to evaluate the interaction between the two tested AA (here, Lys and Thr). This study aimed to simultaneously estimate individual Lys and Thr requirements in pigs fed with daily tailored diets and explore the plasmatic, growth performance, and carcass composition changes associated with differences in PD among pigs.

## 2. Materials and Methods

**Figure 1:**
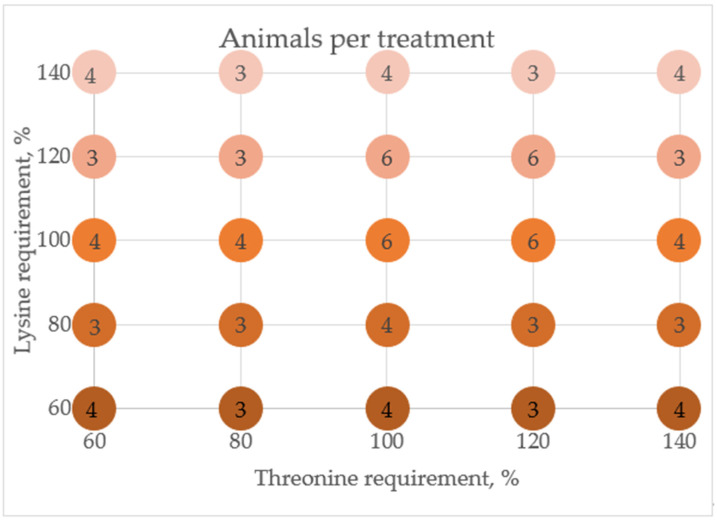
Complete unbalanced factorial design, based on a central composite design, circles contain the number of pigs assigned to each treatment combination of between 60% and 140% threonine and lysine estimated dietary requirements.

A total of 95 growing barrows (Fertilis 25 × G-Performer 8.0; Geneticporc Inc., St-Gilbert, QC, Canada) with high sanitary status were lodged in two 76 m^2^ pens with concrete slats floors of the same mechanically ventilated room. The room temperature was maintained at 22 °C. An electronic chip (Allflex, St-Hyacinthe, QC, Canada) placed in the ear of each pig granted access to the feeders. The adaptation period lasted 14 days, during which they received a commercial feed adjusted to their group nutrient requirements. Water was provided ad libitum with low-pressure nipple drinkers, and feed was provided ad libitum individually throughout the adaptation and the entire 21-day experimental period with ten feeding stations (Automatic and Intelligent Precision Feeder; University of Lleida, Lleida, Spain). The experiment was based on a central composite design using a complete and unbalanced randomized two × five factorial setup including 2 AA (Lys, and Thr) fed at five levels (60%, 80%, 100%, 120%, and 140%) of the estimated requirements. The most extreme treatment combination and outer points were assigned with 4 pigs, intermediate points in the design were assigned with 3, and central points were assigned with 6 (Figure 1). Each pig was considered an experimental unit.

### 2.1. Nutritional Requirements and Diets

Four experimental feeds (A1, A2, A3, and A4; Table 1) were offered to pigs throughout the 21-day experimental period. All AA requirements are expressed in standardized ileal digestible (SID) bases throughout the text. Feeds were formulated to meet 110% of the estimated nutrient requirements, except Lys and Thr, of the most demanding pig at the beginning of the experiment. Therefore, feed A1 was supplemented with crystalline Lys and Thr to satisfy the requirements of the same animals at 140% of the estimated requirements. Similarly, feeds A2, A3 and A4 were supplemented with Lys and Thr at 60% and 140%, respectively, 140% and 60%, and 60% and 60% of the estimated requirements. All feeds were blended daily by the feeders according to the individual requirements of pigs ranging from 60% to 140% Lys and Thr requirements.

Lysine’s daily requirements and the optimal dietary concentration in the blended feed were estimated with the Individual Precision Feeding (IPF) model proposed by Hauschild et al. (2012) using individual daily feed intake and weekly body weight (BW) information. Thus, the empirical component of this model estimates the expected BW, feed intake, and weight gain for the starting day using a time-series approach. The mechanistic model component uses these three latter variables to calculate the optimal concentration of Lys that should be offered that day to each pig to meet its requirements. The mechanistic model component calculated daily Lys requirements (g/d) by adding maintenance and growth requirements. Daily maintenance requirements for Lys were estimated as recommended by van Milgen et al. (2008) by adding basal endogenous losses (0.313 g Lys/kg × daily dry matter intake), losses related to desquamation in the digestive tract (0.0045 g Lys/kg of BW^0.75^ per day), and losses related to the basal renewal of body proteins (0.0239 g Lys/kg of BW^0.75^ per day). Lysine requirements for growth are calculated assuming that 7% of the body protein is Lys [3] and that the efficiency of Lys retention from dietary digestible Lys is 72% [4]. Weight gain composition in terms of protein was calculated assuming 16% protein in daily weight gain [5]. Standardized ileal digestible Thr requirements were calculated using a similar approach to Lys, but the InraPorc model [6] equations for Thr estimation were modified to operate in real-time and incorporated in the IPF model. Daily Thr requirements were estimated by adding basal endogenous losses (0.330 g Thr/kg of daily dry matter intake [7]), losses related to desquamation in the digestive tract (0.0033 g Thr/kg of BW^0.75^ per day [8]), and losses related to the basal renewal of body proteins (0.0138 g Thr/kg of BW^0.75^ per day [8]). Growth requirements for Thr were calculated assuming that 3.7% of the body protein is Thr [9], and that the efficiency of Thr retention from dietary digestible Thr is 61% [6]. Other AA requirements were estimated according to the ideal protein profile concept described by van Milgen and Dourmad [10] and provided such to exceed 10% the maximum requirement when Lys was supplied at 140% of the requirements. Requirements for Thr and Lys were calculated each day for each pig, and AA was provided to each pig according to its assigned Lys and Thr level.

### 2.2. Experimental Measurements

#### 2.2.1. Animal Performance, Nutrient Efficiency, and Carcass Evaluation

Pigs were weighed on arrival and three times during the adaptation period to calibrate the empirical component of model t before the experimental period. Animal performance was evaluated as the average daily feed intake (ADFI), average daily weight gain (ADG), feed-to-gain ratio (G:F), SID Lys intake, SID Thr intake, Lys and Thr efficiency of utilization for PD, PD, the proportion of protein in ADG (PD/ADG, %), and lipid deposition (g/d). Total body fat and lean content were measured by dual X-ray absorptiometry on days 0 and 21 with a densitometer device (GE Lunar Prodigy Advance, Madison, WI, USA). Pigs were scanned in the prone position using the total body scanning mode (Lunar enCORE Software version 8.10.027; Lunar Prodigy Advance, Madison, WI, USA). During the scans, anesthesia was induced with sevoflurane (7%) and maintained with isoflurane (5%).

#### 2.2.2. Blood Sample Collection and Slaughter

Blood samples were collected on days 1 and 21 after 10 h fasting. Blood samples were collected from the jugular vein into a tube containing the anticoagulant EDTA for enzymatic and biochemical analyses or sodium heparin for AA analysis. The time between final sampling and centrifugation of blood samples did not exceed one hour, during which blood samples were kept on ice. Blood samples were centrifuged for 15 min at 1000× *g* at 4 °C. For AA analysis, blood samples were deproteinized within 30 min after centrifugation [11]. All plasma samples were kept at −20 °C during the sampling day and stored at −80 °C until analysis. 

#### 2.2.3. Chemical and Biochemical Analysis

Two replicate of each sample were analyzed following the Association of Official Analytical Chemists (AOAC) standard methods for lyophilization (method 938.18 [12]) and determination of crude protein in the feeds (Kjeltec 2400; FOSS Tecator, Hillerod, Denmark; method 992.15 [12]), lipids (Soxtec 2050 Automated Extraction System; Foss, Höganäs, Sweden; method 991.36; dry matter (method 950.46; AOAC, 1990), and ash (method 920.153 [12]). The AA contents of the samples were measured by gas chromatography coupled to mass spectrometry [13] (Agilent Technologies 7890B gas chromatograph system coupled to an Agilent Technologies 5977A mass selective detector). IgG concentration in blood was determined through ELISA kits (Pig IgG ELISA Quantification Set, ref. E100-104; Bethyl Laboratories, Inc., Montgomery, UK). The biochemical and enzymatic analyses of plasma were performed in an external laboratory (Faculté de médecine vétérinaire, Université de Montréal; Saint-Hyacinthe, QC, Canada) using an automatic analyzer (Beckman Coulter AU680 and AU5800 models, Brea, CA, USA). 

Two pigs per treatment were randomly chosen for slaughter in d 22 and 23, during which treatments were maintained. Pigs were slaughtered in a commercial slaughterhouse, scalded, and scraped, and then the eviscerated carcasses were split longitudinally. The right side of each carcass was dissected, and the head and feet were discarded 24 h after slaughter. The longissimus dorsi muscle was separated from the loin cut and sealed individually in vacuum plastic bags. The bags were transported in a refrigerated truck to the Sherbrooke Research and Development Centre experimental slaughterhouse, where the samples were stored at −20 °C for a maximum of two months. The dissected muscle was cut into cubes, which were then mixed to form a homogeneous pool. The longissimus dorsi muscle was ground four times, and representative subsamples were taken for further analyses. All samples were freeze-dried (method 938.18; AOAC, 1990) and stored at −80 °C until analysis.

### 2.3. Calculations

The total weight gain of pigs was calculated as the difference between the BW measured at the beginning and end of the experimental period. Intake of SID Lys, SID Thr, and crude protein was obtained for each pig by tallying the daily amount of nutrients provided with each of the served feeds. Lysine and Thr efficiency of utilization was calculated by dividing the corresponding amount of available and retained AA. Retention of Lys and Thr were estimated assuming that 7.0% and 3.7% of body protein is Lys and Thr, respectively [6]. The availability of Lys and Thr was estimated by removing the amount used for maintenance from the SID pool. Body lean and fat masses from the scans were converted to their protein and lipid chemical equivalents as proposed by [14]. Protein deposition in gain was calculated by dividing daily PD by the ADG. Protein efficiency and nitrogen excretion were calculated by the difference between the nutrient retained from the respective nutrient intake level. 

### 2.4. Statistical Analysis

Protein deposition as a function of AA intake (Lys and Thr) was analyzed using canonical analysis (RSREG procedure; SAS inc., version 9.4, SAS Inst. Inc., Cary, NC, USA). First, a three-dimensional response surface was generated using nonparametric locally weighted polynomial regression (LOESS procedure). More specifically, the RSREG procedure uses the method of least squares to fit quadratic response surface regression models. The following step was to smooth and model the data using the LOESS procedure of SAS, using a linear and cubic adjustment. These adjustments are the default for this procedure. The LOESS procedure consists of a nonparametric method to estimate regression surfaces by multiple regression analysis. Moreover, this procedure is recommended in the presence of outliers and for data that requires a robust fitting. 

As the Lys and Thr intake explained only a small portion of the variation observed in PD, the factors affecting this variable were studied. Pigs were grouped according to their PD using *k*-means clustering techniques with FASTCLUS procedures of SAS (version 9.4; SAS Inst. Inc., Cary, NC, USA), ignoring treatments. Once pigs were grouped, growth performance, feeding behavior, plasma, and carcass composition in different clusters were compared using a Tukey–Kramer test within the MIXED procedure to consider the unbalanced group size. Based on the results found in this analysis, differences in AA efficiency of utilization were explored. To determine the effect of Lys intake on the Lys efficiency of utilization, pigs receiving Lys between 60 and 100% of the estimated individual Lys requirement were selected (*n* = 32). The condition for selection was that Lys was the first AA limiting, with Thr being provided at least 15% above Lys. The same procedure was adopted to determine the effect of Thr intake on Thr efficiency of utilization (*n* = 26). Estimated individual AA requirements were determined as previously explained (van Milgen et al., 2008; Hauschild et al., 2012). Therefore, AA efficiency of utilization was analyzed as a function of AA intake using the lmer4 package [15] of R (version 3.6.1; R Foundation for Statistical Computing, Vienna, Austria), where the pig was considered the experimental unit and the PD cluster was a random effect. Linear and quadratic effects on the model were tested. Performance package [16] was used to evaluate the models, and the best model was chosen based on the Akaike information criterion (AIC), root Mean Square (RMSE), coefficient of determination (R^2^) for mixed models [17]. The importance of protein clusters to explain AA efficiency of utilization was explored using the inter-correlation coefficient (ICC) [16,17], where adjusted ICC considers the random effects and conditional ICC refers to fixed effects.

## 3. Results and Discussion

### 3.1. The Simultaneous Determination of Two AA Requirements

**Figure 2:**
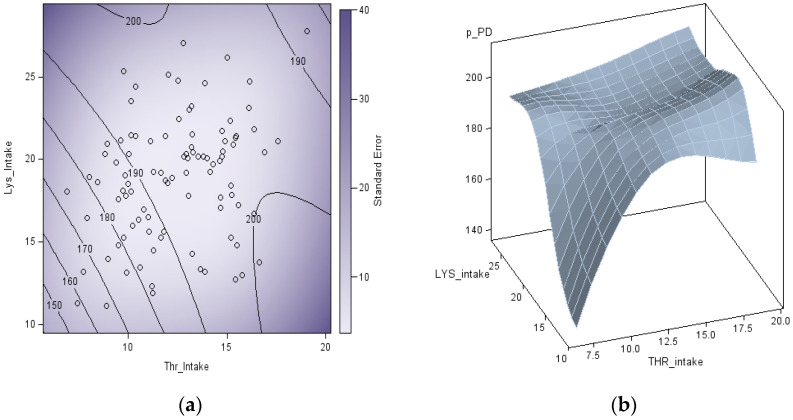
Response contour graphic of the canonical analysis (**a**) of protein deposition (PD; g/day) as a function of lysine intake (g/day) and threonine (g/day) intake, and as three-dimensional response surface (**b**) based on a nonparametric locally polynomial regression method (LOESS function) on a central composite design.

The factorial method used in the present study provided estimates of real-time Lys and Thr requirements. However, these AA requirement estimations had a considerable variation among animals, probably due to the large variation observed in ADG. The predicted response surface observed with the canonical analysis does not provide a unique optimum of Thr and Lys intake, and it is shaped like a saddle (Figure 2a). With a less curved valley orientation of the saddle (eigenvalue of 2.4) aligned with Lys and a curved hill orientation (eigenvalue of −13.6) aligned with Thr. The saddle point indicates that the maximum PD value (199 g/day) resulted from an average of 23 g/day Lys and 14 g/day of Thr intakes. Still, the variation in the response in PD to Lys and Thr observed was large, with Lys and Thr intake explaining only 11% (R^2^ = 0.11) of the variation observed in PD. This large variation in growth performance is often observed in titration trials [11,18,19]. Due to the large data variation and presence of outliers, a more robust adjustment based on the LOESS procedure was adopted. This procedure does not provide parameter estimations for maximum PD or required amounts of Lys and Thr, it was only used to represent the PD response graphically. A cubic adjustment was made to the model (AICC of 7.488; smoothing parameter of 0.9842), resulting in a saddle-shaped surface response (Figure 2b).

D’Mello and Lewis [20] argued that the interdependency of AA hampers an accurate estimation of AA requirements with a dose–response approach as one limiting AA may affect the requirements of the other ones. Different AA combinations are likely possible because pigs have different AA requirements and, likely, different individual AA efficiency. D’Mello and Lewis [20] proposed using a factorial approach to estimate the magnitude of the impact of a change in AA intake and their interaction on AA requirements, instead of determining minimal AA requirements based on the recommendation tables using the dose–response technique. The use of surface response models allows simultaneously quantifying minimum and maximum levels of two factors while considering their interaction [21]. Knowledge on minimal AA requirements combined with knowledge on the magnitude of the impact of AA interaction on AA requirements allows developing a dynamic concept for estimating AA requirements instead of using static AA requirements as proposed in actual factorial methods. It is essential to state that the present study was an exploratory trial, and likely more experimental units per treatment would have allowed for a more straightforward growth response interpretation. The use of central composite design seems to be a viable option for this type of study decreasing the number of experimental units needed [21].

### 3.2. Differences in PD

Ideal protein profiles have been obtained with dose–response studies in which groups of animals are fed with the experimental dietary treatments. In these studies, the optimal level of the tested dietary AA is evaluated in relation to a reference one, usually Lys. The test AA is provided in graded levels, ranging from excess to deficiency, while the reference one is provided at a slightly limiting level (i.e., 90% of the estimated requirements) [10]. Thus, the requirements of the reference AA should be known while all other AA are provided with excesses (i.e., >110%). This approach is used to estimate the ideal protein profile [10]. The among animal variability within treatments is prominent in these studies. It can be hypothesized that in these dose–response studies, the variation of the animals’ response is affected by the variation in the test AA’s requirements and the reference AA. The among pigs PD variation remained large in the present study, and simultaneously estimating two AA (i.e., Lys and Thr) did not reduce this variation. However, variability in the response criterion (i.e., PD) was comparable to that observed in previous swine studies [11,18,22]. Therefore, our results suggest that variability among individual pigs may not be different between the factorial approach used in the present study and the dose–response approach commonly used in swine studies to assess requirements.

**Figure 3:**
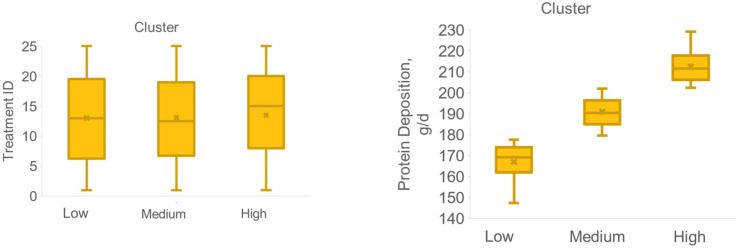
Cluster analysis discriminated pigs with low (167 g/d, *n* = 16), medium (191 g/d, *n* = 38), and high (213 g/d, *n* = 37) protein deposition, presenting no difference in treatment distribution or amino acid intake.

An exploratory analysis was performed to identify the most important factors related to differences in PD response. A cluster analysis was performed ignoring the experimental treatments aiming to understand differences among pigs with different PD. Cluster analysis discriminated pigs with low (167 g/d, *n* = 16, cluster 1), medium (191 g/d, *n* = 38, cluster 2) and high (213 g/d, *n* = 37, cluster 3) PD (Figure 3). Three pigs were excluded from the analysis being outliers after residual evaluation. However, clusters did not differ in Thr and Lys provisions, and treatments were well balanced among clusters (Figure 3).

Furthermore, initial protein and lipid masses, BW, backfat thickness, or loin depth were not different among clustered pigs (Table 2). In terms of growth performance, ADFI, lipid deposition, and backfat and loin depth were similar among clustered pigs. However, ADG, G: F and nitrogen retention increased (*p* < 0.001) as PD increased, and high PD clustered pigs were those presenting the best growth performances. In addition, the proportion (%) of protein in the ADG and the Lys and Thr efficiency of utilization were greater (*p* ≤ 0.05) in the high PD clustered pigs than compared to low PD pigs.

Nonetheless, medium PD clustered pigs did not differ (*p* > 0.05) from low or high PD pigs. High PD pigs presented greater (*p* < 0.05) final BW and body protein mass when compared to low and medium PD pigs. No differences in final longissimus dorsi chemical composition were observed among clusters. As protein AA intake was equal among clusters (Table 3), differences in PD cannot be explained by nutrient supply in the diet. Differences in maintenance could explain it. A large variance (3 to 40%) in the inevitable catabolism of the AA intake was reported by previous studies [4,23,24]. Such differences could explain differences in PD among pigs due to differences in maintenance. Some pigs may maintain high PD because these animals have relatively lower AA catabolism (or maintenance) than animals with lower PD. It has been previously shown that pigs with different PD potential might differ in Lys catabolism [25], contributing to the differences observed in this study.

Final Lys, valine, isoleucine, and homocysteine concentration in plasma (Table 4) were greater (*p* < 0.05) for high PD pigs compared to low PD pigs but did not differ from medium PD pigs. Plasma is the main pool of free AA used in mammals for protein synthesis other than dietary AA. Changes in plasma AA pool concentrations are typically related to changes in other compartments (i.e., liver, muscle) [26]. Homocysteine can be remethylated to methionine or cysteine [27], being a key metabolite in the methionine-cysteine relationship. Given that all pigs ate similar amounts of dietary protein, those having lower PD had probably higher amounts of plasma Met converted to homocysteine; this explains the variation among PD clusters in the concentrations of this metabolite in plasma. Greater concentration of lysine, valine, glycine (product of threonine metabolism), and homocysteine (product of methionine metabolism) in plasma are likely due to the excess of these nutrients given in the diet not being used in their totality by high PD pigs and remained available in plasma. Arginine concentration in plasma was greater (*p* < 0.05) for high PD pigs than for medium PD pigs but did not differ from low PD pigs. Arginine is an essential AA in piglets’ diet, and as animals grow, the intestine contribution synthesizing this AA increases. As high PD pigs were heavier than other clusters, differences in plasma arginine might be due to intestine size.

It is essential to highlight the large amount (50–60% above requirement, Table 2) of branched-chain AAs and tryptophan provided in this experiment. Excesses of leucine [28] and tryptophan [29] might negatively impact the feed intake. In addition, leucine excess might contribute to other branched-chain AA catabolism, which can be avoided by increasing valine in the diet [30]. However, we have not observed changes in plasma AA concentration (Table 3) that could support an imbalance caused by any of these AA excesses. This finding corroborates previous studies establishing that when all the AA are provided in excess, they might correct specific toxic effects of individual AA imbalances [30,31]. Similarly, the sparing effect of branched-chain AA when given in excess on 20% limiting AA [32,33] might have played a role in the low Lys and Thr dietary treatments contributing to the variation in the response.

### 3.3. Differences in the Estimated Lysine and Threonine Efficiency of Utilization

**Figure 4:**
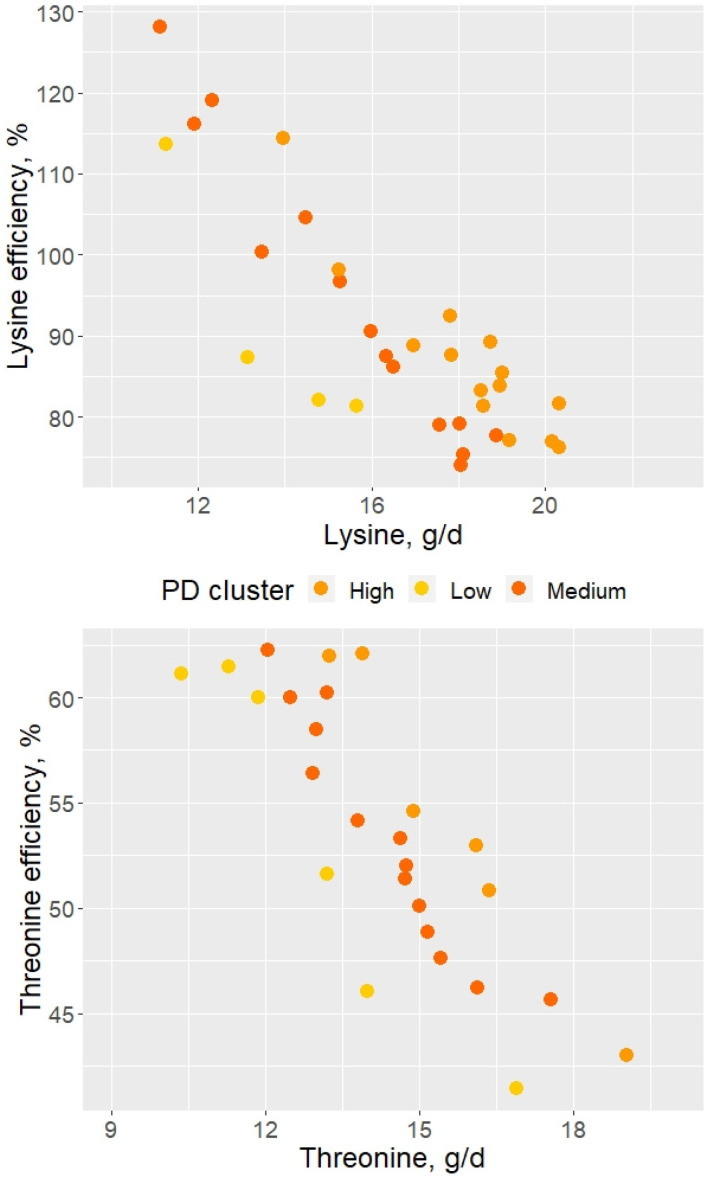
Amino acids efficiency of utilization for protein deposition above maintenance as a function of the amino acid intake.

Lysine and Thr efficiency of utilization decreased (*p* < 0.05) in a quadratic manner (Table 5) as the respective AA intake increased independent of the PD cluster (Figure 4). The current data shows that regardless of the PD potential, animals’ AA efficiency of utilization decreases with increased AA intake. The models showed a quadratic response to AA intake, where the R^2^ marginal (related fixed effect) showed that 74 and 71% of the variance were explained by the Lys and Thr intake, respectively. It is primarily assumed that until the maximal PD is reached Lys is used with the same efficiency, independent of its intake [25]. Nevertheless, when AAs are provided at deficient levels (e.g., 60–70% of the requirements), it seems that the efficiency with which AAs are retained for protein accretion increases [25,34,35]. Amino acid turnover is assumed as a maintenance cost, and that AA originated from protein breakdown will be only used as a source of α-keto acids or even energy in the citric acid cycle. Still, it is estimated that when Lys uptake is critical, 46% to 58% of the Lys from protein turnover can be reused for protein synthesis in the liver [36]. The oxidation of Lys at limiting Lys uptake is critical for its efficiency of utilization for protein deposition [23]. Thus, increases in Lys uptake also result in increases in Lys oxidation, with Lys efficiency of utilization ranging from 85 to 95% in lysine deficient diets [23]. The same study suggests that pigs might cope with low AA provisions actioning mechanisms save the deficient AA. It seems that feeding pigs with daily tailored diets make it possible to target maximal nutrient efficiency by providing nutrients closer to the minimal requirement to sustain growth performance. The last would allow maximizing the use of resources and increase nutrient efficiency of utilization. 

Amino acid intake is not the only determinant of AA efficiency of utilization [23]. In the present study, the ICC shows that 84–87% of the variance in AA efficiency of utilization was between PD clusters, leaving 13 to 16% of the variance within PD clusters. This result means that although individual variance in AA efficiency of utilization exists within the cluster, most of the variance is associated with the difference in PD potential. Furthermore, Moehn et al. [25] observed that decreased catabolism in pigs was determined more by growth potential than by BW or decreased Lys intake. It was previously speculated that increased energy requirements due to increased protein turnover might result in increased variability in performance in situations in which performance deviates from the optimum [37,38]. Still, the protein synthesis in pigs increased linearly and protein breakdown (relative percentage) decreased with the increase of Lys in the diet [39]. This suggests that animals with high PD are more efficient retaining AA or protein than animals with lower PD, in agreement with our calculated AA efficiency presented in this study. Therefore, part of the observed variability in AA requirements among animals might be due to individual differences in energy and protein metabolism, resulting (or causing) differences in the efficiency of AA utilization.

## 4. Conclusions

The factorial approach proposed in the present study allows studying the interaction between the Thr and Lys by avoiding any other AA limiting the response (i.e., the potential impact of any AA other than the test AA on animal response). However, it is important to consider the difficulties associated with this approach, mainly with regard to the statistical power and the biological interpretation of the data. The surface response inherent to the factorial approach used in the present study resulted in a saddle point instead of a unique response for optimal AA requirements. Thus pointing to the possibility of a non-unique response due to the variability in AA requirements among individual pigs, as pigs receiving the same amount of AA might each have a different performance (e.g., different PD). The exploratory cluster analysis performed in this study showed that pigs with greater PD were those with greater gain:feed, ADG and Lys and Thr efficiency of utilization. Around 13% of the variation in the decrease of Lys and Thr efficiency of utilization was associated with increases in AA intake, independent of the PD cluster. More than 80% of the variation in AA efficiency of utilization was explained by the PD potential. These results can contribute to further model individual pigs’ AA efficiency of utilization as a function of PD, and improve AA requirements estimation within precision feeding systems. Best estimations for maximum nutrient efficiency of utilization and growth performance should be studied in a follow-up trial. 

## Figures and Tables

**Table 1:** Feed ingredients and nutrient composition of the experimental feeds A1, A2, A3, and A4.

Item	A1	A2	A3	A4
Ingredient (%)				
Corn	39.81	32.35	38.52	40.42
Wheat	30.00	30.00	30.00	30.00
Canola meal	14.00	14.00	14.00	14.00
Soybean meal	6.20	12.50	6.10	6.10
Soybean oil	3.50	3.90	3.50	3.50
Limestone	1.30	1.28	1.29	1.30
Monocalcium phosphate	0.37	0.32	0.38	0.37
Vitamin-mineral premix ^1^	0.20	0.20	0.20	0.20
Salt	0.53	0.53	0.53	0.53
L-threonine	0.50	0.44	0.00	0.00
L-lysine HCL	0.00	1.26	1.55	0.00
DL-methionine	0.29	0.28	0.34	0.29
L-tryptophan	0.09	0.12	0.16	0.09
L-valine	0.34	0.29	0.41	0.34
L-isoleucine	0.29	0.15	0.26	0.26
L-leucine	0.39	0.23	0.39	0.39
L-histidine	0.15	0.04	0.11	0.15
L-phenylalanine	0.00	0.05	0.17	0.00
L-arginine	0.00	0.00	0.03	0.00
Chemical composition (%)				
Dry matter ^2^	87.27	87.51	87.40	87.21
Crude protein ^2^	15.48	19.00	16.40	15.10
Net energy ^3^ (MJ/kg)	10.25	10.25	10.25	10.24
Crude fiber ^3^	3.62	3.62	3.58	3.63
Calcium ^3^	0.70	0.70	0.70	0.70
Digestible phosphorus ^3^	0.31	0.31	0.31	0.31
Analyzed SID AA ^4^ (%), as-fed basis				
Arginine	0.76	0.95	0.78	0.76
Histidine	0.48	0.43	0.43	0.48
Isoleucine	0.73	0.73	0.73	0.73
Leucine	1.41	1.41	1.41	1.41
Lysine	0.54	1.40	1.40	0.54
Methionine	0.52	0.54	0.57	0.64
Methionine + cysteine	0.79	0.84	0.84	0.88
Phenylalanine	0.60	0.76	0.76	0.57
Threonine	0.94	0.97	0.44	0.44
Tryptophan ^3^	0.24	0.31	0.31	0.24
Valine	0.91	0.98	0.98	0.91

^1^ Vitamin-mineral premix: vitamin A (11,400 IU); vitamin D (1140 IU); vitamin E (35 IU); vitamin K (2 mg); vitamin B12 (30 μg); niacin (20 mg); pantothenic acid (15 mg); pyridoxine (2 mg); thiamine (2 mg); cooper (122 mg); iodine, (0.3 mg); iron (100 mg); manganese (63 mg); selenium (0.3 mg); zinc (152 mg). ^2^ Analyzed values ^3^ Values expected base on diet composition. ^4^ Standardized ileal digestible (SID) were estimated from the analyzed total amino acid and crude energy content in the feed, and values from estimated total and SID values provided by the formulation software Brill Formulation^®^ (© 2017 Cargill, Incorporated. All Rights Reserved), total amino acid values were analyzed using GC-MS.

**Table 2:** Body composition and growth performance of growing barrows (35–60 kg body weight) fed daily tailored diets varying lysine (Lys) and threonine (Thr) levels and clustered in low, medium, and high protein deposition (PD).

Item	Low PD	Medium PD	High PD	MSE ^1^	*p*-Value
Number of observations	16	38	37		
Initial body conditions					
Body weight, kg	35.8	34.5	34.7	2.11	0.14
Lipids, g	1793	1698	1684	195	0.17
Protein, g	7225	6966	6999	422	0.11
Body protein, %	20.2	20.2	20.2	0.19	0.85
Backfat thickness, cm	6.5	6.2	6.3	0.82	0.56
Loin depth, cm	35.9	37.0	36.4	2.66	0.36
Growth performance ^2^					
ADFI, kg	1.93	1.91	1.89	0.06	0.83
ADG, kg	0.97 ^a^	1.06 ^b^	1.16 ^c^	0.06	<0.0001
Gain:feed	0.51 ^a^	0.56 ^b^	0.62 ^c^	0.06	<0.0001
PD, g/d	167 ^a^	191 ^b^	213 ^c^	7.42	<0.0001
Lipid deposition, g/d	171	160	166	42.62	0.64
PD in the ADG, %	17.3 ^a^	18.0 ^ab^	18.3 ^b^	0.90	0.00
Nitrogen retained, %	50 ^a^	57 ^b^	64 ^c^	7.42	<0.0001
Thr ^3^ efficiency, %	59.0 ^a^	64.3 ^ab^	69.9 ^b^	14.91	0.05
Lys ^3^ efficiency, %	70.2 ^a^	74.0 ^ab^	80.8 ^b^	14.51	0.04
Loin deposition, mm	12	11	13	3.32	0.30
Backfat deposition, mm	2.0	2.7	2.5	1.08	0.10
Number of meals per day	12	11	11	2.86	0.57
Final body conditions					
BW, Kg	56.0 ^a^	56.8 ^a^	59.1 ^b^	2.8	<0.001
Lipids, g	5391.9	5054.4	5160.0	258.1	0.55
Protein, g	10,730 ^a^	10,976 ^a^	11,462 ^b^	105.0	<0.0001
Backfat thickness, cm	8.41	9.07	8.86	0.34	0.27
Loin depth, cm	48.3	48.3	49.0	1.04	0.72
Longissimus dorsi muscle *					
Crude protein, %	22.69 ^a^	22.46 ^ab^	22.41 ^b^	0.10	0.08
Lipids, %	1.49	1.36	1.44	0.12	0.64
Collagen, %	1.28	1.25	1.29	0.03	0.37
Lys, g/100 g of CP	9.2	9.3	9.5	0.51	0.42
Thr, g/100 g of CP	4.5	4.6	4.6	0.06	0.56

* *n* = 9, 16, 20 in cluster low, medium, and high, respectively; ^1^ MSE, maximum standard error. ^2^ ADFI = averaged daily feed intake, ADG = average daily gain, ^3^ Lysine and threonine efficiency = amino acid efficiency of utilization for protein deposition above maintenance, where retention of Lys and Thr were estimated assuming that 6.9% of body protein is Lys, and 3.7% is Thr. ^a^^–c^ Values within a row without a common letter differ according to the Tukey test.

**Table 3:** Average nutrient intake in growing barrows (35–60 kg body weight) fed daily tailored diets varying lysine (Lys) and threonine (Thr) levels, and clustered in low (167 g/d), medium (191 g/d), and high (213 g/d) protein deposition (PD).

Item	Low PD	Medium PD	High PD	MSE ^1^	*p*-Value
Number of observations	16	38	37		
Crude protein, g/d	337.6	336.7	336.3	10.15	0.99
Lys, g/d	17.8	18.7	19.6	3.73	0.24
Thr, g/d	12.0	12.4	12.9	2.61	0.48
Methionine, g/d	11.1	10.8	10.6	0.39	0.53
Tryptophan, g/d	5.4	5.5	5.5	0.17	0.88
Valine, g/d	17.0	17.0	16.9	2.10	0.97
Leucine, g/d	24.0	24.2	24.3	2.91	0.93
Isoleucine, g/d	13.3	13.2	13.1	1.67	0.90
Phenylalanine, g/d	12.9	13.0	13.1	0.40	0.94
Feed A, %	11.2	11.8	9.6	4.77	0.88
Feed B, %	33.1	39.3	48.1	7.71	0.22
Feed C, %	26.5	28.5	28.1	6.6	0.97
Feed D, %	29.2 ^a^	20.4 ^ab^	14.2 ^b^	4.6	0.03

^1^ MSE, maximum standard error. ^a,b^ Values within a row without a common letter differ according to the Tukey test.

**Table 4:** Final plasma amino acid concentration (μmol) in growing barrows (35–60 kg body weight) fed daily tailored diets varying lysine (Lys) and threonine (Thr) levels and clustered in low (167 g/d), medium (191 g/d), and high (213 g/d) protein deposition (PD).

Item	Low	Medium	High	MSE ^1^	*p*-Value
Number of observation	16	38	37		
Lysine	144.0 ^a^	166.9 ^ab^	169.9 ^b^	10.3	0.10
Threonine	139.5	149.6	159.7	11.3	0.31
Methionine	35.4	36.3	37.8	2.1	0.60
Tryptophan	54.5	58.8	55.0	2.2	0.12
Valine	343.7 ^a^	374.4 ^b^	358.2 ^ab^	9.9	0.03
Leucine	183.5	199.3	189.6	7.1	0.13
Isoleucine	110.6 ^a^	121.2 ^b^	115.4 ^ab^	4.2	0.08
Phenylalanine	69.8	68.3	66.4	2.7	0.55
Glycine	867.7 ^a^	971.5 ^b^	976.6 ^b^	37.1	0.04
Serine	91.5	98.3	95.3	3.2	0.19
Cysteine	227.2	234.4	231.1	4.0	0.29
Alanine	274.8	275.5	253.5	16.8	0.32
Arginine	147.4 ^ab^	160.8 ^a^	145.8 ^b^	6.6	0.04
Asparagine	31.0	33.2	31.9	1.0	0.14
Aspartic acid	7.1	7.3	7.2	0.5	0.94
Glutamate	110.2	111.0	103.4	8.5	0.60
Glutamine	421.8	439.9	458.0	15.0	0.12
Homocysteine	26.1 ^a^	28.7 ^ab^	31.1 ^b^	1.2	26.1
Proline	168.54	172.46	178.28	5.08	0.23
Tyrosine	69.98	73.08	71.96	2.90	0.67

^1^ MSE, maximum standard error. ^a,b^ Values within a row without a common letter differ according to the Tukey test.

**Table 5:** Mixed model coefficients used to explore the variance in Lysine (Lys) and Threonine (Thr) efficiency of utilization above maintenance as a function of the amino acid intake using a Bayesian approach, where the protein deposition cluster was considered a random effect.

	Lysine Efficiency		Threonine Efficiency	
Item	Model 1 (SE)	Model 2 (SE)	Model 3 (SE)	Model 4 (SE)
Intercept	188.729 (8.081) **	274.589 (23.862) **	102.400 (3.720) **	127.548 (15.673) **
Linear slope-Lys intake	−6.183 (0.366) **	−17.505 (3.020) **		
Quadratic slope-Lys intake		0.362 (0.096) **		
Linear slope-Thr intake			−3.405 (0.206) **	−6.897 (2.129) **
Quadratic slope-Thr intake				0.119 (0.072) *
AIC ^1^	204.583	194.975	124.45	123.93
BIC	210.446	202.304	129.482	130.22
Num. observations	32	32	26	26
Num. groups: cluster	3	3	3	3
Variance cluster (random intercept)	92.256	85.163	14.97	16.369
Variance residual	19.042	12.876	3.413	3.025
R^2^ conditional (fixed and random effect)	0.95	0.97	0.95	0.96
R^2^ marginal (fixed effect)	0.72	0.74	0.72	0.71
RMSE	4.16	3.42	1.74	1.64
ICC	0.83	0.87	0.81	0.84

* *p*-value <0.05; ** *p*-value <0.01. ^1^ AIC = Akaike information criterion, RMSE = root Mean Square, R^2^ = coefficient of determination [16], ICC = inter correlation coefficient [16,17].

## Data Availability

The datasets generated and/or analyzed during the current study belong to Her Majesty the Queen in Right of Canada, as represented by the Minister of Agriculture and Agri-Food Canada, and are not publicly available. Data can be obtained from the authors upon reasonable request and with the permission of the representative of Her Majesty the Queen in Right of Canada.
